# Interleukin-21 Receptor Signalling Is Important for Innate Immune Protection against HSV-2 Infections

**DOI:** 10.1371/journal.pone.0081790

**Published:** 2013-12-16

**Authors:** Sine K. Kratholm, Marie B. Iversen, Line Reinert, Simon K. Jensen, Marianne Hokland, Thomas Andersen, Andrew Rankin, Deborah Young, Sebastian Frische, Søren R. Paludan, Christian K. Holm

**Affiliations:** 1 Department of Biomedicine, Aarhus University, Aarhus, Denmark; 2 Immunology and Autoimmunity, Pfizer Inc. Cambridge, Massachusetts, United States of America; 3 The Water and Salt Research Centre, Institute of Anatomy, Aarhus University, Aarhus, Denmark; McMaster University, Canada

## Abstract

Interleukin (IL) -21 is produced by Natural Killer T (NKT) cells and CD4^+^ T cells and is produced in response to virus infections, where IL-21 has been shown to be essential in adaptive immune responses. Cells from the innate immune system such as Natural Killer (NK) cells and macrophages are also important in immune protection against virus. These cells express the IL-21 receptor (IL-21R) and respond to IL-21 with increased cytotoxicity and cytokine production. Currently, however it is not known whether IL-21 plays a significant role in innate immune responses to virus infections. The purpose of this study was to investigate the role of IL-21 and IL-21R in the innate immune response to a virus infection. We used C57BL/6 wild type (WT) and IL-21R knock out (KO) mice in a murine vaginal Herpes Simplex Virus type 2 (HSV-2) infection model to show that IL-21 – IL-21R signalling is indeed important in innate immune responses against HSV-2. We found that the IL-21R was expressed in the vaginal epithelium in uninfected (u.i) WT mice, and expression increased early after HSV-2 infection. IL-21R KO mice exhibited increased vaginal viral titers on day 2 and 3 post infection (p.i.) and subsequently developed significantly higher disease scores and a lower survival rate compared to WT mice. In addition, WT mice infected with HSV-2 receiving intra-vaginal pre-treatment with murine recombinant IL-21 (mIL-21) had decreased vaginal viral titers on day 2 p.i., significantly lower disease scores, and a higher survival rate compared to infected untreated WT controls. Collectively our data demonstrate the novel finding that the IL-21R plays a critical role in regulating innate immune responses against HSV-2 infection.

## Introduction

HSV-2 is an enveloped double stranded DNA virus [1] that replicates lytically in permissive cells of the epithelial linage causing mucocutaneous lesions as seen in genital herpes infections in humans [2]. HSV-2 is also a neurotropic virus with the ability to establish latent infection in sensory neurons from where the infection can be reactivated [1]. HSV-2 infection can also cause more severe disease e.g. neonatal herpes, which has a high mortality rate [1], [3], [4].

Defence against HSV-2 infection involves both innate and adaptive immunity. Epithelia cover all external and internal surfaces of the body, comprising the first physical and chemical barrier of the innate immune system to invading pathogens [5]. NK cells constitute an important part of the early innate immune defence against herpes virus infections, and humans lacking NK cells are highly susceptible to herpes infections [6], [7]. This is also the case in murine models of herpes virus infections as Thapa et al. showed that viral titers in the vagina and central nervous system (CNS) are higher in NK cell depleted mice challenged with a vaginal HSV-2 infection [8]. Further, Ashkar et al. found that mice lacking NK cells are highly susceptible to vaginal HSV-2 infection [9]. Central to NK cell derived anti-viral activity is the ability of NK cells to kill infected cells and to produce IFN-γ [10], [11].

IL-21 and the IL-21R were first described in year 2000 by two independent groups [12], [13]. IL-21 is produced by activated CD4^+^ T-cells [12] and NKT cells [14] and signals through a heterodimer class 1 type cytokine receptor consisting of the IL-21R_α_-chain in complex with the common cytokine receptor γ-chain (γ_c_-chain) [15], [16]. IL-21 is the only cytokine known to signal through the IL-21R [12]. The functional heterodimer receptor is expressed in lymphoid tissue such as the spleen, thymus and lymph nodes by most lymphoid and myeloid cells from both the innate and the adaptive immune system [12], [14], [17]-[19] but also by epithelial cells and fibroblasts [20]-[22]. Expression of the γ_c_-chain in non-immune cells has previously been reported in epithelial cells [23], [24]. The effects of IL-21 in innate and adaptive immunity are pleiotropic and include enhanced proliferation of lymphoid cells, increased cytotoxicity of CD8^+^ T cells and NK cells and differentiation of B cells into plasma cells [19], [25], [26]. Conversely IL-21 can also inhibit maturation and antigen-presentation by dendritic cells and can be pro-apoptotic for B cells and NK cells in a context dependent manner [17], [27], [28].

We have previously found that IL-21 mRNA levels were increased in splenic CD4^+^ T cells during a systemic HSV-2 infection from day 3-7 p.i. [29]. The role of IL-21 in the immune response to virus was further highlighted as Elsaesser et al. showed that IL-21R KO mice are unable to clear a lymphocytic choriomeningitis virus infection that causes a chronic viral infection due to decreased ability to sustain T cell function [30]. Other studies also conclude that IL-21 is important for the adaptive immune system to control chronic viral infection [31]–[33].

Little attention has been paid to the effect IL-21 could have in innate immunity to virus infections. Cells belonging to the innate immune system such as epithelial cells, NK, NKT cells and macrophages express the functional heterodimer IL-21R and respond to IL-21 [14], [18]–[20], [22], thus they are potential targets for IL-21 in the innate stages of a viral infection (day 1-3 p.i.). IL-21 induces expression of genes important in activating innate immune responses [34] and *in vitro* and *in vivo* studies show that NK cells proliferate and increase cytotoxicity in response to IL-21 [19], [35]. Activated NK and NKT cells are important in the early defence against virus infection [6], [9], [11] and *in vitro* NKT cells can produce substantial amounts of IL-21 [14].

In this study we investigated the role of IL-21 and the IL-21R in innate immunity to virus infections using a murine vaginal HSV-2 infection model. Following vaginal inoculation, HSV-2 replicate in the vaginal epithelial cells giving rise to local inflammation. Virus then spreads to the CNS, via sensory nerve endings that innervate the vagina, causing myelitis and neurological symptoms [36]. We report that IL-21R expression was present in the vagina of u.i. WT mice and expression increased significantly in the vaginal tissue on day 1-3 p.i. with HSV-2. The IL-21R was expressed by the vaginal epithelia and the increase in IL-21R expression was due to increased receptor expression in the vaginal epithelial cells, suggesting a role for IL-21 signalling through the IL-21R at this early and innate stage of infection. We were able to support this as mice lacking the IL-21R had elevated vaginal viral titers on day 2 and 3 p.i. and adoptive transfer of WT splenocytes was not able to rescue IL-21R KO mice. In addition, treatment with mIL-21 resulted in decreased vaginal viral titers after intra-vaginal HSV-2 infection also in the innate stages of infection. Absence of the IL-21R did not affect NK cell recruitment to the infected vagina, nor alter IFN-γ production or degranulation activity in vaginal NK cells from infected mice. In conclusion, the data presented here demonstrate that the IL-21R is important for innate immune protection against HSV-2 infections.

## Materials and Methods

### Ethics statement

All animal experiments in this study were approved by Danish Government authorities, (The Ministry of Food, Agriculture and Fisheries) and comply with Danish law (permit # 2009/561-1613). Mice were monitored 3 times daily during infection and all efforts were made to minimize suffering.

### Reagents

Ischoves Modified Dulbeccos Medium (IMDM) supplemented with glutamine, hepes and NaHCO_3_ (pH  =  7.2). Eagles Minimal Essential Medium (MEM) supplemented with 2% glutamine, antibiotics (200 IU/mL penicillin, 200 µg/mL streptomycin, 0.5% nystatin and 0.1% gentamycin) 10% NaHCO_3_ and 2% or 5% heat inactivated Fetal Calf Serum (FCS) (BioWittaker). RPMI 1640 growth media (BioWittaker) was supplemented with 1% hepes (pH  =  7.1), antibiotics (200 IU/mL penicillin, 200 µg/mL streptomycin), 1% glutamine and 10% FCS.

### Mice

We used specific pathogen-free 8–10 weeks old, age matched, female C57BL/6 (Taconic M&B, Denmark) and IL-21R KO mice on C57BL/6 background. To evaluate the distribution of splenocytes in the adoptive transfer experiment we used congenic female C57BL/B6.Cg-Igha Thy1a Gpi1a/Crl (Thy1.1^+^, Charles River Laboratories, Brussels, Belgium) and C57BL/6JBomTac mice (Thy1.2^+^, Taconic M & B, Ry, Denmark). Mice were housed in the animal facility at Aarhus University.

### Murine intra-vaginal HSV-2 infection model

Mice were pre-treated with 2 mg of Depo-Provera (Pfizer) injected subcutaneously. Five days later mice were anaesthetized with isoflurane (Baxter) and inoculated vaginally with 6.7×10^4^ PFUs (plaque forming units) HSV-2 suspended in 20 µL of IMDM. Mice were kept under anaesthesia and placed on their backs for 10 min. Mice were monitored and scored daily for genital inflammation, neurological symptoms and death. Disease severity was graded by the following score: 0) Healthy 1) Genital erythema, 2) Moderate genital inflammation, 3) Purulent genital inflammation and bad general condition (lack of activity, pilo erection), 4) Hind limb paralysis. Mice were sacrificed by cervical dislocation when reaching score 4. mIL-21 treated mice were inoculated vaginally with 5 µg mIL-21 suspended in 20 µL of PBS on day -1, 0, 1, 2 and 3. Infected untreated controls and u.i. controls received 20 µL of PBS (vehicle). On day 1, 2 and 3 p.i. vaginal fluids were collected by pipetting 40 µL of IMDM in and out of the vagina 12 times, repeated twice. Washes were put on dry ice immediately and stored at –70 °C until analysis. In separate experiments mice were euthanized and the vaginas were harvested on day 1, 2 and 3 p.i respectively for RNA extraction. In other experiments vaginas were harvested on day 1 p.i. for immunohistochemistry (IHC) or on day 2 p.i. for flow cytometry.

### Immunohistochemistry of murine vagina for light microscopy

The vaginal tissue was fixed by applying 2% formaldehyde in PBS through the vaginal opening. The vaginal tissue was dissected out and placed in similar fixative for 24 hrs. and subsequently dehydrated and embedded in paraffin. The paraffin-embedded tissues were sectioned and immunolabelled as previously described [37]. IL-21R primary antibodies (AbCam product # 13268) and HRP-linked goat anti-rabbit secondary antibodies (P448, DAKO) were used. Light microscopy was carried out using a Zeiss light microscope (Zeiss, Brock & Michelsen A/S, Denmark).

### Quantitative RT-PCR

RNA from vaginal tissue was extracted with TRIzol (Invitrogen) according to the manufactureŕs recommendations. Briefly vaginal tissue was homogenized in TRIzol and chloroform was added, followed by phase separation by centrifugation. RNA was precipitated with isopropanol and pelleted by centrifugation. Pellets were washed in 80% ethanol and dissolved in RNase-free water. Contaminating DNA was removed by DNase 1 (Ambion) treatment. IL-21, IL-21R and β-Actin mRNA was quantified using TaqMan Gene Expression Assay (Applied Biosystems) according to manufacturer’s recommendations. Expression of mRNA for Pathogen Recognition Receptors (PRRs) comprising Toll like receptor (TLR)2, TLR3, TLR9, DNA-dependent activator of IFN-regulatory factors (DAI), retinoic acid-inducible gene 1 (RIG-I), melanoma differentiation-associated protein 5 (MDA-5), absent in melanoma 2 (AIM-2) and IFN-γ inducible protein 16 (IFI 16) in vaginal tissue of u.i. mice was quantified using SYBR-green (Stratagene).

### Virus and vaginal viral titers

For *in vivo* intra-vaginal infection we used HSV-2 strain 333. Vaginal viral titers on day 1, 2 and 3 p.i. were determined by standard plaque assays on monolayers of Vero cells. Vero cells were plated in petri dishes with 1.25×10^6^ cells per dish in 5 mL MEM enriched with 5% FCS. Cells were incubated over night at 37 °C and 6% CO_2_. On day 2 media was discarded and 100 µL of samples in serial dilution were added together with 400 µL of MEM supplemented with 2% FCS and incubated for 1 hr. Every 15 min. the tissue culture plates were rocked to ensure even distribution of the virus. After 1 hr. 8 mL of MEM supplemented with 2% FCS and 0.2% human immunoglobulin (CLS Behring) was added. Cells were incubated for 2 days, stained with 0.03% methylene blue and plaques were counted.

### IFN-α/β bio-assay

IFN-α/β bioactivity was measured by a L929 cell-based bio-assay. In brief samples of vaginal washes or murine IFN-α/β as standard were plated in a 96 well plate in successive 2-fold dilution. Virus in the samples was killed by UV-light treatment for 6 min. 2×10^4^ L929 cells/well in 100 µL MEM supplemented with 5% FCS were added. Plates were incubated over night at 37 °C. On day 2 vesicular stomatitis virus (VSV/V10) was added to the wells. Plates were incubated for 2 days. The dilution mediating 50% protection was defined as 1 U/mL of IFN-α/β.

### Cytokines

Levels of IL-6, IL-10, IL-12p70, IFN**-**γ, TNF-α, MCP-1 (CCL-2) and KC (CXCL-1, murine homologue of IL-8) protein in vaginal washes were measured by Luminex Technology using kits from Bio-Rad. IL-21 was measured by Ready-SET-Go ELISA kit (eBioscience).

### Fluorescence Activated Cell Sorting (FACS) and flow cytometry

Single-cell suspensions of vaginal cells were obtained by mechanic and enzymatic treatment. Vaginas were cut into small pieces using a scalpel, suspended in 4 mL Collagenase/Dispase (1 mg/mL) (Roche) and 2 mL DNase 1 (2 mg/mL) (Roche) and incubated for 45min. at 37 °C under constant gentle shaking. For intra-cellular staining monensin (Bio Legend) was added. Cell suspensions were filtered through a mesh with a pore size of first 70 µm, followed by 40 µm to remove debris. Enzymes were inactivated by 10 mL of PBS with 0.02% EDTA. Cells were pelleted at 300×g for 8 min. and washed with PBS. Prior to staining cells were blocked for 10 min. at room temperature (RT) with anti-mouse CD16/CD32 (clone 93, eBioscience) and mouse IgG (Jackson Immunoresearch Inc.). For sorting, cells were stained for 30 min. at RT with anti-mouse CD45-APC (clone 30-F11, BD Biosciences) and dead cells were identified by propidium iodide (PI). Sorted CD45^+^ cells were used in the cytotoxicity assay (see bellow). For flow cytometry the following anti-bodies were used: anti-mouse CD4-PE (clone RM4-5) and anti-mouse Thy1.2-APC (clone 53-2.1), anti-mouse NK1.1-PE and anti-mouse NK1.1-APC (clone PK 136), anti-mouse CD107a+b-FITC (clone ID4B, ABL-93), anti-mouse IFN-γ-PE (clone XMG1.2), anti-mouse CD314-PE (clone CX5), anti-mouse CD-25-PE (clone 3C7) all from BD Biosciences and anti-mouse granzyme-B-FITC (clone NGZB, eBioscience). LIVE/DEAD marker (Invitrogen) was used to identify dead cells. Before intra-cellular staining, cells were permeabilized with Perm2 buffer (BD Biosciences). Immediately before flow cytometry, Flow-Count beads (Beckman Coulter, Fullerton, CA) were added. Cell sorting and flow cytometry was performed on a FACSAria III (BD Biosciences) and a FC500 (Beckman Coulter) respectively. Data were analysed with FlowJo version 9.5.3.

### Cytotoxicity assay

CD45^+^ FACS sorted vaginal cells were cultured in the absence or presence of MHC class 1 deficient YAC-1 cells in a ratio of 2:1 (vaginal cells:YAC-1 cells) for 3 hrs. in RPMI 1640 with 10% FCS, monensin and anti-mouse CD107a+b-FITC. After incubation cells were stained with anti-mouse NK1.1-PE for 30 min. at RT and washed in PBS before analysis.

### Adoptive transfer of splenocytes

Spleens were harvested from 10 weeks old WT and IL-21R KO mice. Single cell suspensions were made from the spleens using a glass grinder. Cells were suspended in PBS and counted in tryphan blue. At harvest more than 98% of splenocytes were viable. Each mouse (IL-21R KO) received 20×10^6^ cells (injected intraperitoneally (i.p.)) harvested from either WT or IL-21R KO mice. The day after the transfer of splenocytes, mice were infected with HSV-2 as described above. To examine the distribution of adoptively transferred splenocytes we used Thy1.1 and Thy1.2 mice. Thy1.1 mice were injected i.p. with 20×10^6^ purified CD4+ cells from Thy1.2 mice. One day after injection, Thy1.1 mice were sacrificed and the spleens were evaluated for the presence of adoptively transferred cells. Single cell suspensions were made as described above. Cells were stained with rat-anti-mouse Thy1.2-APC conjugated and rat-anti-mouse CD4-PE conjugated. Immediately before flow cytometry, Flow-Count beads (Beckman Coulter, Fullerton, CA) were added.

### Statistics

Statistical analysis was performed using the software Prism 5.0d (Graph Pad Software, San Diego CA). Statistical analysis was determined by Mann-Whitney unpaired test or by Log-Rank (Mantel Cox) test for survival. *p*-values < 0.05 were considered to be statistically significant. Statistical analysis has been performed on all data sets. Differences reaching statistical significance are marked with asterisk’s (* *p*<0.05, ** *p*<0.01, *** *p*<0.001) in the figures.

## Results

### IL-21R is expressed in vaginal tissue in u.i. and HSV-2 infected WT mice on day 1-3 p.i

**Figure 1:**
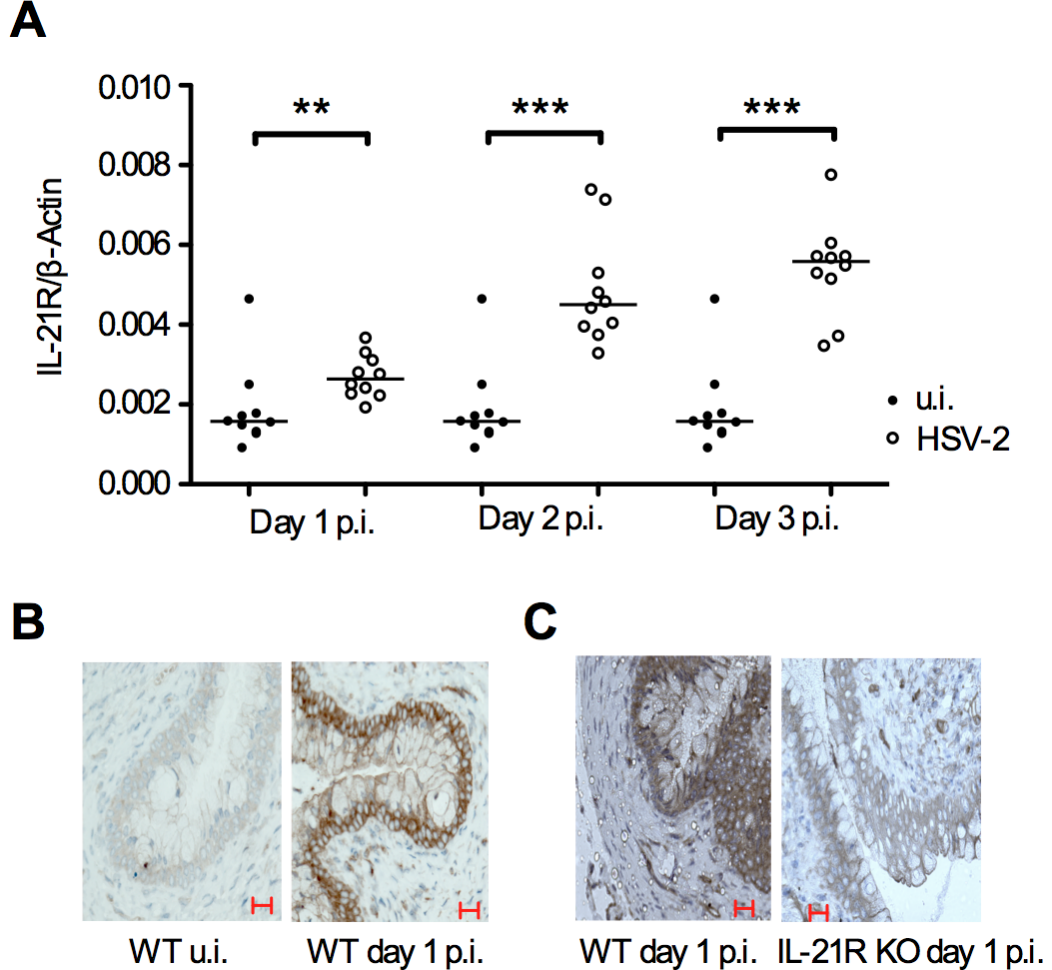
IL-21R is expressed by vaginal epithelial cells in WT mice and IL-21R expression is increased by HSV-2 infection. 8 weeks old C57BL/6 mice were infected intra-vaginally with 6.7×10^4^ PFUs HSV-2 and vaginas were harvested on day 1-3 post infection (p.i.) (**A**) Quantitative RT-PCR analysis of mRNA encoding IL-21R in vaginal tissue in uninfected (u.i.) and HSV-2 infected WT mice on day 1-3 p.i. (**B-C**) IHC staining with IL-21R primary antibodies and HRP-linked goat anti-rabbit secondary antibodies on 2 µm sections of vaginal tissue in (**B**) u.i. and HSV-2 infected WT mice day 1 p.i. and in (**C**) HSV-2 infected WT and IL-21R KO mice day 1 p.i. as control. Scale bar  =  20 µm. (**A**) IL-21R is normalized to β-Actin in each sample. Horizontal bars indicate median. Each dot represents one animal. Data are representative of two individual experiments (*n*  =  10 mice per group respectively). ** *p*<0.01, *** *p*<0.001.

To test whether IL-21 could be involved in the innate immune response to HSV-2 infections we investigated IL-21 and IL-21R expression at the site of infection on day 1-3 p.i. We measured IL-21 protein in vaginal washes and IL-21 and IL-21R mRNA levels in vaginal tissue from C57BL/6 WT mice infected intra-vaginally with HSV-2 and from u.i. controls. In addition we did IHC staining for IL-21R protein in vaginal tissue. We found that IL-21R mRNA was expressed in the vagina in u.i. WT mice and that expression increased significantly in the vagina after HSV-2 infection compared to u.i controls on day 1, 2 and 3 p.i. (Figure 1A). IL-21R was expressed by the vaginal epithelia in u.i. mice and the expression in the epithelia increased after HSV-2 infection (Figure 1B-C). Levels of IL-21 protein and mRNA were below the limit of detection (data not shown). These data show that IL-21R expression is present in the epithelia of the u.i vagina, and increased in the vaginal epithelia during the innate phase of the immune response to HSV-2 infection.

### IL-21R is important for the innate anti-viral response

**Figure 2:**
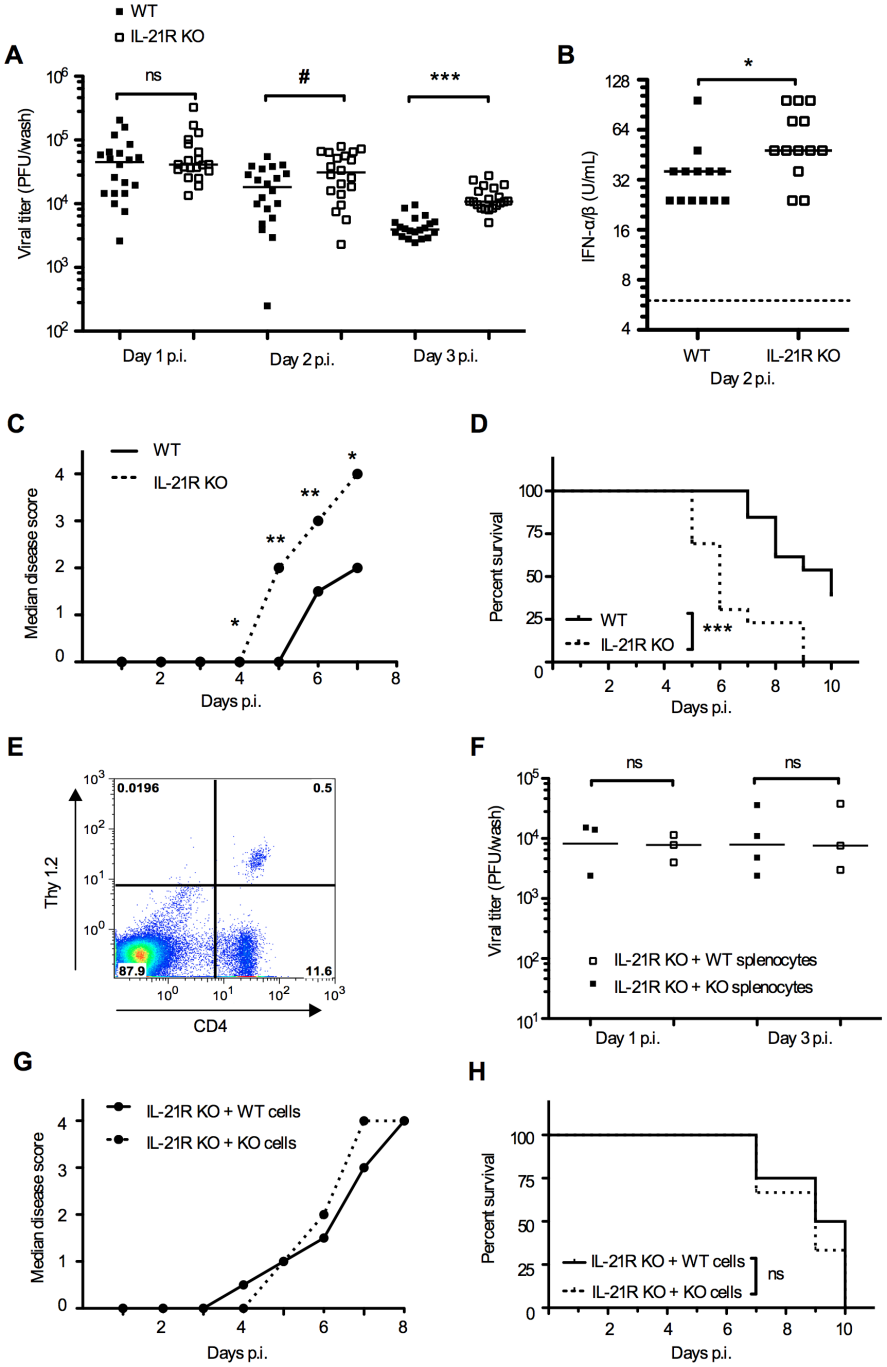
Mice lacking the IL-21R are more susceptible to HSV-2 infection than WT mice. (**A-D**) 10 weeks old mice were infected intra-vaginally with 6.7×10^4^ PFUs HSV-2. Vaginal fluids were collected on day 1-3 p.i. for quantification of (**A**) vaginal viral load day 1-3 p.i. and (**B**) IFN-α/β protein levels in vaginal fluids day 2 p.i. The dashed line indicates the lower detection limit of the assay. (**C**) After HSV-2 infection mice were scored daily for disease development. Data are shown as median disease scores for WT and IL-21R KO mice. (**D**) When reaching score 4 (hind limb paralysis) mice were euthanized and recorded. Data are shown in a Kaplan Meier plot of survival, *p*<0.001. (**A-B**) Horizontal bars indicate median. Each dot represents one animal. (**A-D**) Data are representative of two independent experiments (*n*  =  7 and 13 mice per group respectively, in A data are pooled). * *p*<0.05, ** *p*<0.01, *** *p*<0.001, # *p*  =  0.058 when using a Mann-Whitney test but when applying an unpaired Students t-test *p*  =  0.02. ns  =  not significant. (**E**) 20×10^6^ CD4^+^ splenocytes from Thy1.2 mice were adoptively transferred i.p. to Thy1.1 mice. Spleens were harvested the following day and splenocytes were analysed by flow cytometry for Thy1.2 and CD4. (**F-H**) 10 weeks old IL-21R KO mice were injected i.p. with 20×10^6^ splenocytes from 10 weeks old WT or IL-21R KO mice respectively. One day after the transfer, mice were infected intra-vaginally with 6.7×10^4^ PFUs HSV-2. (**F**) Vaginal fluids were collected on day 1-3 p.i. for quantification of vaginal viral load day 1-3 p.i. Horizontal bars indicate median. Each dot represents one animal. (**G**) After HSV-2 infection mice were scored daily for disease development. Data are shown as median disease scores for IL-21R KO mice transferred with WT or IL-21R KO splenocytes. Differences in median disease score between the two groups did not reach statistical significance on any of the days. (**H**) When reaching score 4 (hind limb paralysis) mice were euthanized and recorded. Data are shown in a Kaplan Meier plot of survival. ns  =  not significant. Data (**E-H**) represent one experiment.

To investigate the importance of the IL-21R in the innate anti-viral immune response we infected IL-21R KO and WT mice with HSV-2 and measured viral titers and IFN-α/β production in the innate phase. Vaginal fluids were collected on day 1-3 p.i. and analysed for viral content and IFN-α/β. At day 2 and 3 p.i. vaginal viral titers were significantly higher in IL-21R KO mice compared to WT (Figure 2A). Moreover, IFN-α/β levels were significantly higher in the IL-21R KO mice compared to WT on day 2 p.i. (Figure 2B). IFN-α/β was also measured on day 1 and 3 p.i. but data is not shown since IFN-α/β is detectable in the vagina after intra-vaginal HSV-2 infection on day 2 only and not day 1 and 3 p.i. [38]. The same kinetic is also seen with IFN-γ [9], [10], [39]. Mice were also monitored and scored for genital inflammation, neurological symptoms and death. The median disease scores in IL-21R KO mice were significantly higher from day 4-7 p.i. compared to WT mice (Figure 2C). Furthermore, IL-21R KO mice had a significantly lower survival rate following HSV-2 infection compared to WT mice (*p*<0.001) (Figure 2D). To investigate further if the increased susceptibility could be due to the lack of IL-21R on cells of the adaptive immune system we conducted an experiment with adoptive transfer of either WT or IL-21R KO splenic leukocytes (primarily B and T cells) into IL-21R KO mice. Mice were subsequently infected intra-vaginally with HSV-2. When analysing viral titers in vaginal washes we found no difference between the groups that received WT and IL-21R KO cells respectively (Figure 2F). Mice were also monitored and scored for genital inflammation, neurological symptoms and death. We observed no difference in median disease score or survival rate (Figure 2G-H). To examine the distribution of adoptively transferred splenocytes in the recipient mice, we made a control experiment were we transferred CD4^+^ splenocytes from Thy1.2 mice to Thy1.1 mice. The day after the adoptive transfer we harvested the spleens from the Thy1.1 mice and analysed splenocytes by flow cytometry, stained with anti-mouse CD4-PE and anti-mouse Thy1.2-APC. We were able to detect the adoptively transferred CD4^+^ Thy1.2^+^ cells in the spleen (Figure 2E, upper right quadrant). In conclusion adoptive transfer of splenocytes from WT mice was not sufficient to rescue the IL-21R KO mice. This supports that higher susceptibility to HSV-2 in the IL-21R KO mice depended on non-adaptive immune mechanisms.

**Figure 3:**
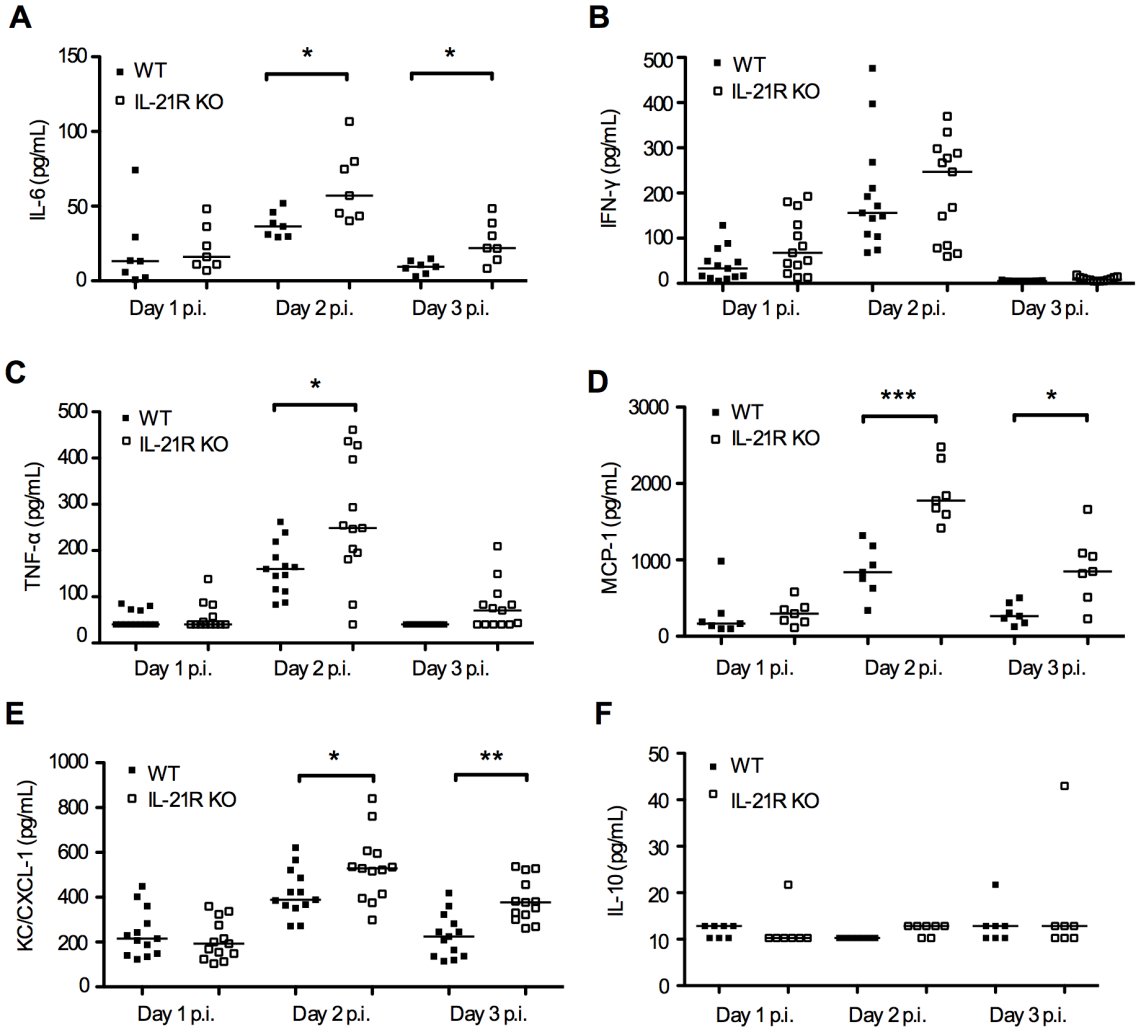
Cytokine levels in vaginal fluids from HSV-2 infected IL-21R KO and WT mice. Vaginal fluids were collected on day 1-3 p.i. with 6.7×10^4^ PFUs HSV-2 intra-vaginally. (**A-F**) IL-6, IFN-γ, TNF-α, MCP-1 (CCL-2), KC (CXCL-1, murine homologue of IL-8) and IL-10 protein levels in vaginal fluids from HSV-2 infected IL-21R KO and WT mice, quantified by Luminex. Horizontal bars indicate median. Each dot represents one animal. Data are representative of two individual experiments (*n*  =  7 and 13, 10 weeks old mice, per group respectively). Statistical analysis has been performed on all data sets. Where differences between WT and IL-21R KO mice reached statistical significance it is indicated in the graph by * *p*<0.05, ** *p*<0.01, *** *p*<0.001. When differences did not reach statistical significance there is no indication in the graph.

In addition we quantified vaginal cytokine levels in IL-21R KO and WT mice, including IL-6, IL-10, IL-12p70, IFN**-**γ, TNF-α, MCP-1 (CCL-2) and KC (CXCL-1). These are all important cytokines in the innate response to HSV-2 infections [40], [41]. IL-6, MCP-1 and KC levels were significantly elevated in vaginal washes on both day 2 and 3 p.i. and TNF-α was elevated on day 2 p.i. in IL-21R KO mice compared to WT (Figure 3A-E). IL-12p70 was not detectable (data not shown) while IL-10 levels were close to or below the detection limit in both groups (Figure 3F). To determine if the increased susceptibility to viral infection of the IL-21R KO mice could be due to an inherent altered expression of PRRs we measured mRNA levels of TLR2, TLR3, TLR9, MDA-5, AIM-2, DAI, RIG-I and IFI16. All are known to be activated by HSV-2 [42]. We found no difference in PRR expression in vaginal tissue from u.i. WT, IL-21R KO mice and WT mice pre-treated with mIL-21 (data not shown) thus suggesting that lack of IL-21R or addition of mIL-21 did not alter the capability of sensing and responding to viral Pathogen Associated Molecular Patterns (PAMP’s). Together, these data show that IL-21 – IL-21R signalling is important in the innate immune response to a vaginal HSV-2 infection.

### mIL-21 treatment has anti-viral effect in the innate immune response to HSV-2 infection

**Figure 4:**
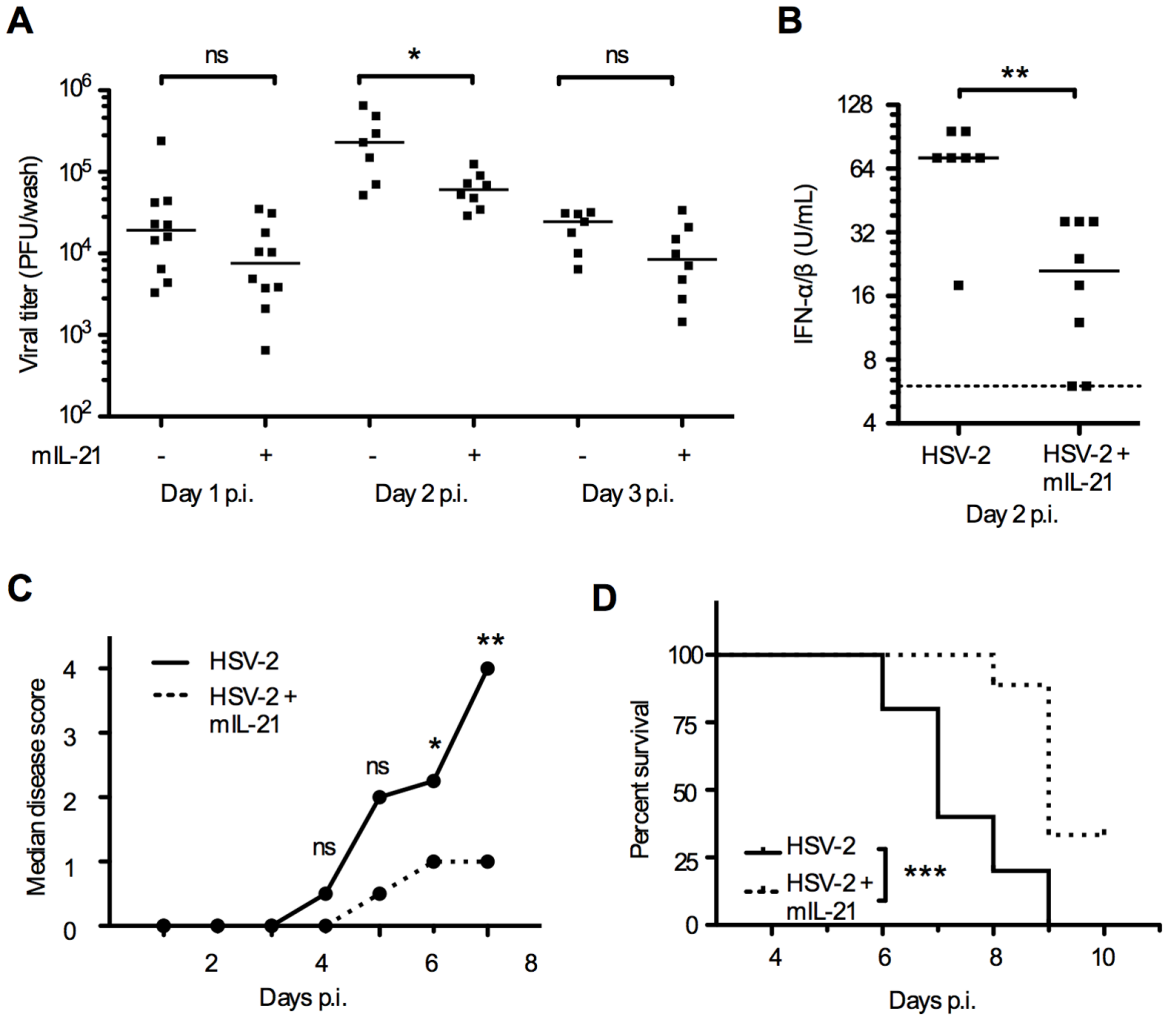
mIL-21 treatment has anti-viral effect in the innate immune response. 8 weeks old WT mice were infected intra-vaginally with 6.7×10^4^ PFUs HSV-2. mIL-21 treated mice were inoculated intra-vaginally with 5 µg mIL-21 on day -1 to 3 p.i., and untreated mice received PBS. Vaginal fluids from WT mice infected with HSV-2 and untreated (-) or treated (+) with mIL-21, were collected on day 1-3 p.i. for quantification of (**A**) vaginal viral load day 1-3 p.i. and (**B**) IFN-α/β protein levels in vaginal fluids day 2 p.i. The dashed line indicates the lower detection limit of the assay. (**C**) After HSV-2 infection mice were scored daily for disease development. Data are shown as median disease scores in HSV-2 infected, untreated or mIL-21 treated WT mice. (**D**) When reaching score 4 (hind limb paralysis) mice were euthanized and recorded. Data are shown in a Kaplan Meier plot of survival, p<0.001. (**A-B**) Horizontal bars indicate median. Each dot represents one animal. (**A-D**) Data are representative of three individual experiments (*n*  =  10 mice per group). * *p*<0.05, ** *p*<0.01. ns  =  not significant.

**Figure 5:**
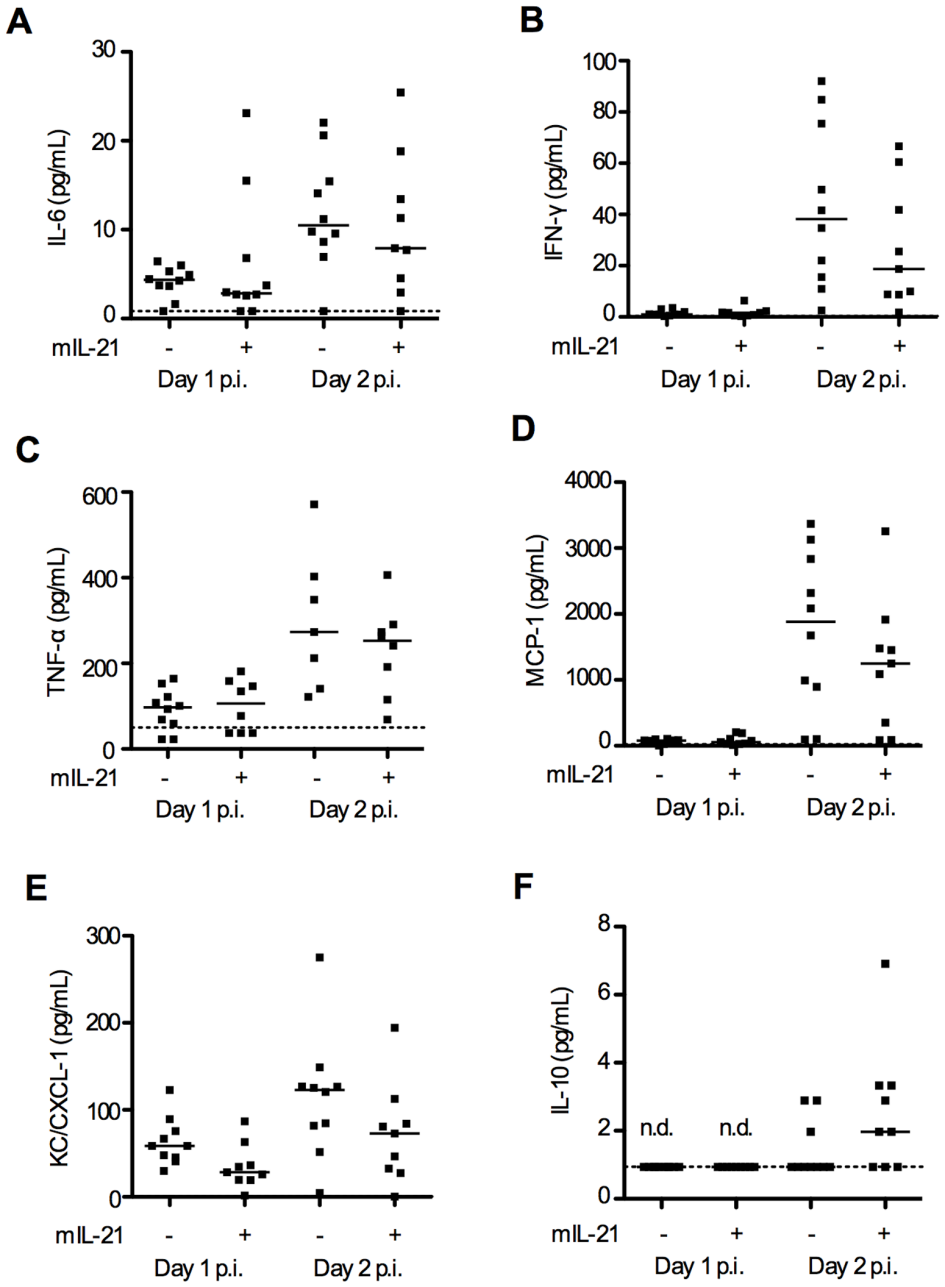
Cytokine levels in vaginal fluids from mIL-21 treated or untreated, HSV-2 infected WT mice. Mice were infected intra-vaginally with 6.7×10^4^ PFUs HSV-2. mIL-21 treated mice (+) were inoculated intra-vaginally with 5 µg mIL-21 on day -1 to 3 p.i., and untreated mice (-) received PBS. Vaginal fluids were collected on day 1 and 2 p.i. (**A-F**) IL-6, IFN-γ, TNF-α, MCP-1 (CCL-2), KC (CXCL-1, murine homologue of IL-8) and IL-10 protein levels in vaginal fluids on day 1 and 2 p.i. with HSV-2, quantified by Luminex. Horizontal bars indicate median. Each dot represents one animal. Dashed lines indicate lower detection limit of the assay. Data are representative of three individual experiments (*n*  =  10, 8 weeks old mice, per group). n.d. not detectable. Statistical analysis has been performed on all data sets. Differences in cytokine levels between mIL-21 treated and untreated mice did not reach statistical significance.

As the IL-21R expression was increased in vaginal tissue early after HSV-2 infection (Figure 1A-B) and disease was more severe in IL-21R KO mice than in WT (Figure 2) we concluded that the IL-21 - IL-21R network is important in the innate response to genital herpes in mice. Given the critical role of the IL-21R in regulating the innate immune response to HSV-2 we next sought to determine the impact of exogenous IL-21 treatment on the course of HSV-2 infection in the intra-vaginal HSV-2 infection model. WT mice were treated intra-vaginally with mIL-21 or vehicle on day -1, 0, 1, 2 and 3 p.i. Mice receiving mIL-21 treatment had a significantly lower median vaginal viral titer on day 2 p.i. compared to untreated infected mice (Figure 4A). Median vaginal viral titers were also lower on day 1 and 3 p.i. in mIL-21 treated mice although it did not reach statistical significance (Figure 4A). In addition, the IFN-α/β levels in vaginal washes were significantly lower in mIL-21 treated mice day 2 p.i. (Figure 4B). Moreover, mIL-21 treated mice developed less genital inflammation than untreated controls. The median disease scores were higher in untreated infected mice compared to mIL-21 treated mice from day 3-7 p.i. and significantly higher on day 6 and 7 p.i. (Figure 4C). mIL-21 treated mice also showed a significantly increased survival compared to HSV-2 infected untreated controls (Figure 4D). The IL-6, IL-10, IL-12p70, IFN**-**γ, TNF-α, MCP-1 (CCL-2) and KC (CXCL-1) levels in vaginal fluids from mIL-21 treated and untreated HSV-2 infected mice were measured. mIL-21 treatment did not lead to altered levels of pro-inflammatory cytokines on day 1, 2 (Figure 5A-E) or 3 p.i. (data not shown). We found a small but not significant increase in IL-10 levels in mIL-21 treated mice on day 2 p.i. (Figure 5F). Again, we were unable to detect IL-12p70. We conclude that mIL-21 treatment of mice has anti-viral effect in the innate stages of the immune response to vaginal HSV-2 infection.

### Vaginal NK cell effector activity in IL-21R KO and WT mice

**Figure 6:**
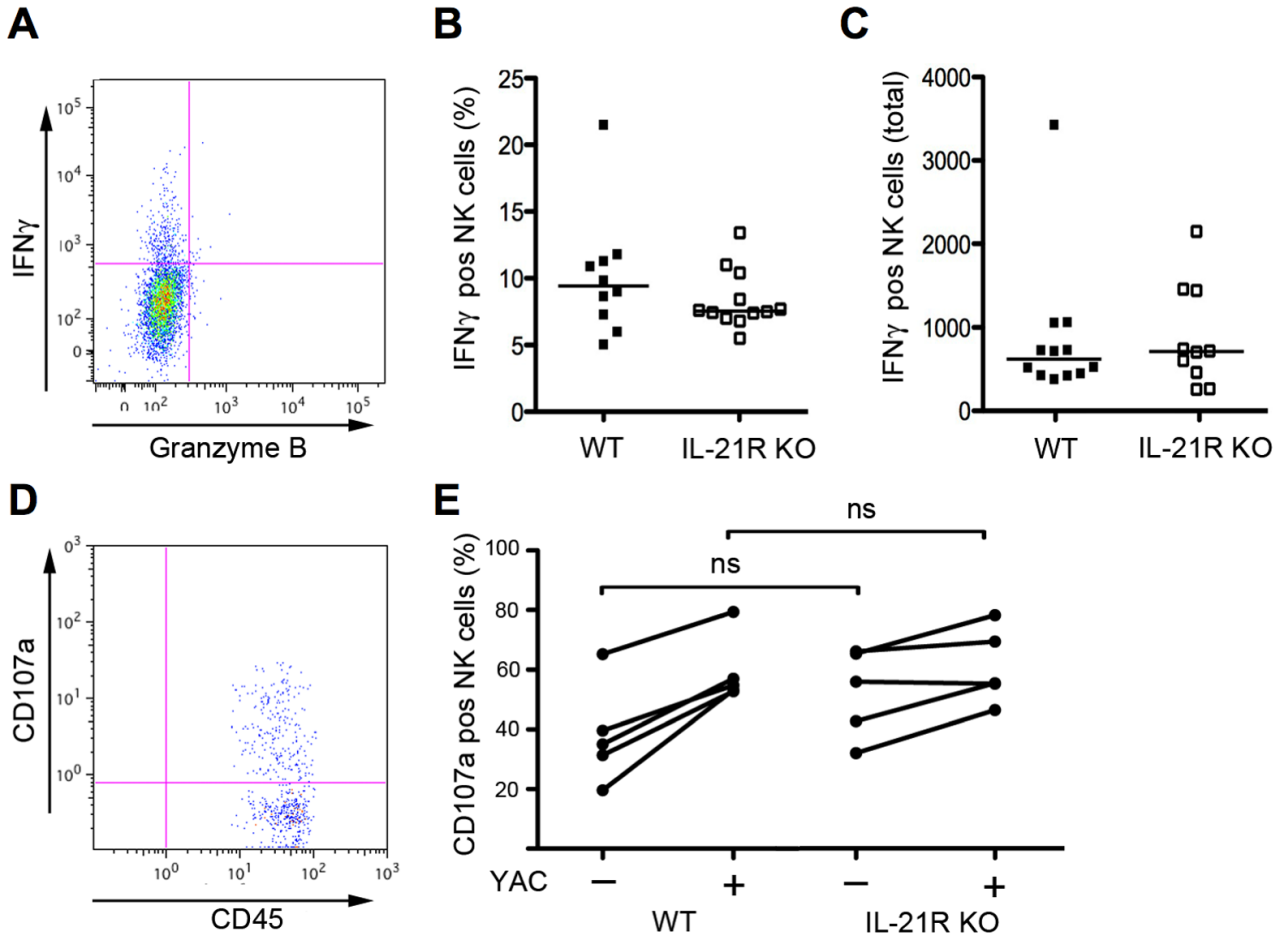
Vaginal NK cell effector activity in IL-21R KO and WT mice day 2 p.i. with HSV-2. Vaginas were harvested on day 2 p.i. with 6.7×10^4^ PFUs HSV-2 intra-vaginally. (**A**) Example of intracellular IFN-γ and granzyme B staining in vaginal NK cells. (**B**) IFN-γ^+^ vaginal NK cells in % per mouse in IL-21R KO and WT mice. (**C**) Total number of IFN-γ^+^ vaginal NK cells per mouse in IL-21R KO and WT mice. (**D**) CD107a+b expression on FACS sorted viable CD45^+^ vaginal cells from WT mice, cultured in the presence or absence of MHC class 1 deficient YAC-1 cells, and gated on NK cell marker NK1.1. (**E**) CD107a+b^+^ NK cells in %, of FACS sorted viable CD45^+^ vaginal cells, cultured in the absence or presence of YAC-1 cells, and gated on NK1.1, in IL-21R KO and WT mice. (**A-C**) *n*  =  12 mice per group. (**D-E**) *n*  =  6 mice per group. (**B-C**) Horizontal bars indicate median. Each dot represents one animal. All mice were 8-9 weeks old. ns  =  not significant.

NK cells are important in innate anti-viral defence and they express the functional heterodimer IL-21R [6], [19]. As IL-21R KO mice had higher vaginal viral titers than WT mice (Figure 2A), we addressed the mechanisms responsible for these differences in vaginal viral titers. Milligan et al. showed that the level of IFN-γ in vaginal fluids is biphasic with a peak on day 2 p.i. This peak disappears when NK cells are depleted [10]. By flow cytometry, we measured intra-cellular IFN-γ and granzyme B in vaginal NK cells isolated day 2 p.i. There was no difference in relative or absolute numbers of NK cells recruited to the vagina between WT and IL-21R KO mice after HSV-2 infection (data not shown). We did not detect a difference in neither the relative, nor the total number of IFN-γ^+^ vaginal NK cells per mouse, between IL-21R KO and WT mice (Figure 6A-C). Very few cells stained positive for granzyme B and these were detected in similar amounts in both WT and IL-21R KO mice (data not shown). Next we tested the ability of vaginal NK cells to degranulate as a measure of NK cell cytolytic activity. We FACS-sorted vaginal CD45^+^ cells from infected WT and IL-21R KO mice day 2 p.i. These cells were then cultured either in the presence or absence of the NK cell sensitive cell line YAC-1. Cells were then stained for the degranulation markers CD107a+b and subsequently analysed by flow cytometry. We found no significant difference in NK cell CD107a+b surface expression between IL-21R KO and WT mice (Figure 6D-E). In conclusion, there was no difference in NK cell recruitment to the vagina, and NK cells showed unaltered production of IFN-γ and unaltered ability to degranulate between WT and IL-21R KO mice on day 2 p.i. where viral titers were increased in IL-21R KO compared to WT mice.

## Discussion

We have studied the importance of IL-21 and the IL-21R in the innate immune response against HSV-2 infections in a murine intra-vaginal HSV-2 infection model. Our data demonstrate the novel finding that IL-21R signalling is important in innate immune protection against HSV-2 infections. This adds to the current knowledge that IL-21 is necessary to sustain CD8^+^ T cell effector activity in chronic viral infections, making IL-21 an important cytokine in anti-viral immunity [30]-[32]. We found the IL-21R to be important for control of viral replication in the vagina during the innate phases of the immune response to HSV-2 and subsequently to limit disease progression.

Milligan et al. investigated T cell-mediated mechanisms in a murine vaginal HSV-2 infection model and found that HSV-2 specific T cells are present in the draining genital lymph nodes from day 4 p.i. and in the urogenital tract from day 5 p.i. [43]. Moreover T cell derived IFN-γ is found in vaginal fluids only from day 4-8 p.i. [10]. Similar kinetics are demonstrated by Rakasz et al. [44]. To confirm that the phenotype in the IL-21R KO mouse after HSV-2 infection (Figure 2) depended on innate immune mechanisms we conducted an experiment with two groups of IL-21R KO mice receiving adoptive transfer of splenocytes from either WT mice or from IL-21R KO mice as control. Mice were subsequently infected intra-vaginally with HSV-2. We found no difference in vaginal viral titers on day 1-3 p.i. (Figure 2F), nor did we observe any difference in median disease score or survival rate (Figure 2G-H). Adoptive transfer of immune cells from WT mice to IL-21R KO mice were not sufficient to rescue the IL-21R KO mice, supporting that higher susceptibility to HSV-2 in the IL-21R KO mice depended on early innate immune mechanisms. Therefore we conclude that the effects of IL-21 on vaginal viral titers, type I IFN and cytokines on days 1-3 p.i. depended on innate immune mechanisms. This is further supported by the high expression levels of IL-21R in the epithelial cells lining the vaginal lumen on day 1 p.i.

We examined expression of PRŔs known to be activated by HSV-2 infection [42], in vaginal tissue from u.i WT, IL-21R KO and mIL-21 treated WT mice to determine if the increased susceptibility to HSV-2 infection of the IL-21R KO mice could be due to an inherent altered expression of PRRs. We found no difference in PRR expression showing that lack of IL-21R signalling or addition of mIL-21 did not affect the PRR expression, and thus suggesting that lack of IL-21R or addition of mIL-21 did not alter the capability of sensing and responding to viral Pathogen Associated Molecular Patterns (PAMP’s).

In our study, we showed that infected IL-21R KO mice expressed higher levels of IFN-α/β and IL-6, TNF-α, MCP-1 and KC, when compared to infected WT controls. These increases are in line with the higher vaginal viral load found in the IL-21R KO mice. HSV-2 PAMP’s activate different PRR’s (TLR2, TLR3, TLR9, MDA-5, AIM-2, DAI, RIG-I and IFI16) [42]. Activation of these PRR’s induces intracellular signalling pathways that lead to the expression of proteins with pro-inflammatory and anti-microbial activity, such as IFN-α/β and cytokines. A higher viral load thus activates more PRR’s resulting in an increased expression of IFN-α/β and cytokines [42].

We were unable to detect IL-21 in the vaginal tissue in both u.i and HSV-2 infected WT mice. This could be explained by IL-21 being produced by a relatively small number of cells and by the poor sensitivity of currently available assays for detection of IL-21 protein. A plausible candidate to supply IL-21 during the innate immune response is NKT cells. Data published by Ashkar et al. indicate that NKT cells are present in the vagina in the innate phase of a vaginal HSV-2 infection [9] and Coquet et al. show that NKT cells can produce significant amounts of IL-21 after *in vitro* stimulation [14]. Future research should focus on detecting the source of IL-21 production during innate anti-viral immune responses.

The functional heterodimer IL-21R is highly expressed by cells from the innate immune system [12], [14], [17]–[19] and IL-21 is known to increase IFN-γ production and cytotoxicity of NK cells [19], which are important effector cells in early control of viral infections [6]. In fact, Milligan et al. showed that NK and NKT cell derived IFN-γ enhances resolution of genital HSV-2 infection [10]. IFN-γ production from innate sources peaks on day 2 p.i. and disappears when NK cells are depleted [10]. We addressed the mechanism responsible for the differences in vaginal viral titers that we found between HSV-2 infected IL-21R KO and WT mice by quantifying relative and absolute numbers of IFN-γ producing NK cells in the vagina on day 2 p.i. in IL-21R KO and WT mice. However we found no difference between the two groups. This is in line with the finding that there was no difference in IFN-γ protein levels in vaginal fluids day 2 p.i. between IL-21R KO and WT mice (Figure 3B). Next we addressed the cytolytic activity of vaginal NK cells measured as their ability to degranualte, but found no difference in NK cell CD107a+b surface expression between IL-21R KO and WT mice. These results are in accordance with results from Kasaian et al. concluding that IL-21R KO mice have normal numbers of NK cells that are fully able to respond to activating agents such as poly(I:C) and IL-15. Coquet et al. have reported an IL-21 mediated effect on NK cell receptor expression on NKT cells. We examined expression of CD314 (NKG2D) and CD25 on NK cells in the vagina on day 2 p.i. in IL-21R KO and WT mice but found no difference (data not shown). Additionally, lack of IL-21R signalling had no effect on the number of NK cells recruited to the infection site, leading us to conclude that the effect of IL-21 and IL-21R on viral replication, type I IFN and cytokine production was not NK cell dependent.

Apart from cells of the immune system, IL-21R expression has also been demonstrated in non-lymphoid cells such as epithelial cells and fibroblasts, and increased expression has been shown in keratinocytes from patients with systemic sclerosis and in gut epithelial cells from patients with inflammatory bowel disease [20]–[22]. Expression of the γ_c_-chain in non-immune cells such as epithelial cells has previously been reported [23], [24]. We showed that vaginal epithelial cells in u.i WT mice expressed the IL-21R and that IL-21R expression in the epithelium increased early after HSV-2 infection. Caruso et al. have shown that IL-21R expression is increased by IL-21 but not by other cytokines such as IL-6, IL-1β, IFN-γ or TNF-α [20]. The increased expression of IL-21R in the vagina after HSV-2 infection supports that IL-21 is induced at the innate stages of infection. The functions of IL-21R on epithelial cells are unknown, but stimulation of these cells with IL-21 could contribute to decreased susceptibility to infection. Future research in this area could focus on unravelling how IL-21 affects epithelial cells during infections and.

In conclusion, our data show that the IL-21 – IL-21R network is important in the innate stages of the immune response to HSV-2 infection. Moreover, treatment with mIL-21 has anti-viral effects during the innate phase of the immune response to HSV-2. Therefore, in addition to the role in adaptive immune responses to virus, we demonstrate that IL-21 and the IL-21R also has an important role in innate immune protection against HSV-2 infection.
